# Qualitative assessment of online information about age-related macular degeneration available in Portuguese

**DOI:** 10.1590/S1679-45082018AO4240

**Published:** 2018-05-25

**Authors:** Jorge Agi, Niro Kasahara, Claudio Luiz Lottenberg

**Affiliations:** 1 Santa Casa de Misericórdia de São Paulo, São Paulo, SP, Brazil; 2 Hospital Israelita Albert Einstein, São Paulo, SP, Brazil

**Keywords:** Information, Macular degeneration, Internet, Quality control, Patient education, Age effect, Informação, Degeneração macular, Internet, Controle de qualidade, Educação de pacientes, Efeito idade

## Abstract

**Objective:**

To evaluate the quality of online information on age-related macular degeneration available in Portuguese.

**Methods:**

The search term “age-related macular degeneration” was used to browse the web using four different search engines. The first 40 websites appearing on match lists provided by each search engine were recorded and those listed in at least three tab pages selected. The Sandvik Severity Index was used as to assess website quality.

**Results:**

Quality of information available on selected websites was rated average (mean Sandvik Score 7.08±2.23).

**Conclusion:**

Most websites disseminating information about age-related macular degeneration were of average quality. The need to readjust web-based information to target lay public and promote increased understanding was emphasized.

## RESUMO

**Objetivo:**

Avaliar a qualidade das informações sobre degeneração macular relacionada à idade, em português, disponi bilizadas na internet.

**Métodos:**

Utilizou-se a palavra-chave “degeneração macular relacionada à idade” em quatro buscadores para a pesquisa na internet. Os 40 primeiros *sites* que apareceram em cada buscador foram registrados, e aqueles que apareceram em pelo menos três buscadores foram incluídos no estudo. Como ferramenta para a avaliação da qualidade, foi utilizado o *Sandvik Score*.

**Resultados:**

A informação disponibilizada nos *sites* selecionados apresentou qualidade mediana (escore médio de Sandvik 7,08±2,23).

**Conclusão:**

A maioria dos *sites* na internet sobre degeneração macular relacionada à idade apresentou qualidade mediana, refletindo a necessidade de readequação da informação difundida, para aproximá-la do público leigo.

## INTRODUCTION

Age-related macular degeneration (AMD) is a chronic degenerative disease affecting the central portion of the retina. The condition is often bilateral and associated with vision loss. Two clinical presentations have been described: exsudative (humid) and non-exsudative (dry). Visual impairment resulting from AMD may prevent affected individuals from reading, writing or driving, with significant impacts on patients’ quality of life.^(^ ^1^ ^)^

Advanced stages of AMD are debilitating. However, some forms of the disease can be successfully controlled with current therapies, allowing patients to maintain or even partially recover vision.^(^ ^2^ ^,^ ^3^ ^)^

Age-related macular degeneration is the leading cause of blindness in older adults in developed countries. Its prevalence increases with age and 11.5% of white individuals aged over 80 years are thought to be affected.^(^ ^1^ ^)^ Early AMD is characterized by the presence of many very small (less than 64µ in diameter) or some intermediate (64 to 124µ in diameter) drusen, while several intermediate or large-sized (125µ or larger in diameter) drusen may be seen in intermediate stages of the disease.^(^ ^4^ ^)^ Advanced AMD is defined by geographic atrophy or choroidal neovascularization.^(^ ^5^ ^)^ A two-fold increase in the number of early/intermediate AMD cases is expected to occur over the next decades, as life expectancy increases.^(^ ^6^ ^,^ ^7^ ^)^ In the United States, the prevalence of early/intermediate or advanced AMD in individuals aged 40 years or older was 5.7% and 0.8%, respectively, in 2008.^(^ ^6^ ^)^ In Brazil, approximately three million individuals aged over 65 years are thought to suffer from varying stages of AMD.^(^ ^8^ ^)^

The epidemiological significance of AMD has raised research interest and led to the development of several therapeutic strategies aimed to prevent and/or halt the progression of such a severe ocular condition.^(^ ^9^ ^)^

Aside from potential blindness, AMD is associated with stigmatization of patients who still lead an active life, and is a major public health concern.^(^ ^2^ ^,^ ^3^ ^)^ Therefore, growing efforts are being made to increase population awareness and encourage the individuals to seek ophthalmological assessment.^(^ ^2^ ^,^ ^8^ ^,^ ^10^ ^)^

The search for information about AMD and related consequences has increased significantly over the last few years, with the internet representing the primary search tool. The internet now has over one billion users and, according to an European study, more than 60% of adult population looks for health and medical information online.^(^ ^11^ ^)^ However, quality control over information disseminated on the internet is fragile at best.

The advent of the internet has brought about profound changes in patient-healthcare professional relationship, in terms of information on health and disease, once almost exclusively available to such professionals and now widely disseminated in this virtual environment.^(^ ^12^ ^)^ In the new reality introduced by the recent internet phenomenon, in which physicians are increasingly confronted with patients who bring data collected online, quality of information has become a central concern within the medical community. False information disseminated online may create confusion among those relying on electronic sources.

Several Brazilian websites disseminate medical information to the lay public and no clear quality control mechanisms have been put in place so far. Quality of information material is directly correlated with schooling levels in the general population, given the significant contribution of socioeducational background to the understanding of information.

To date, no studies investigating the quality of AMD information disseminated on Brazilian websites have been undertaken.

## OBJECTIVE

To investigate the quality of information on age-related macular degeneration available in Portuguese, on the internet.

## METHODS

A quantitative study investigating the quality of AMD information available on systematically sampled websites from March 25 to March 29 2017. This project did not require approval by the Ethics in Human Research Committee of the *Irmandade da Santa Casa de Misericórdia de São Paulo* (project number, 9117).

### Website identification and selection

This study was based on systematic web search for information in the Portuguese language. The term “age-related macular degeneration” was searched using the following search engines: Google, Bing, Yahoo Search and ASK. The first 40 websites appearing on each match list (*i.e.*, the first four web pages comprising 10 websites each) were recorded. Websites simultaneously located by at least three search engines were selected. The full content of selected web pages was evaluated, excluding links.

### Qualitative assessment of web-based information

The qualitative assessment of selected websites was performed by two examiners following discussion and consensus of opinion about results obtained using the Sandvik Score.^(^ ^13^ ^)^ In this tool, quality levels are scored according to the following criteria: ownership, authorship, publication period, source, interactivity, web navigation and content balance. Each criterion is scored from zero to 2, as follows: ownership (2 – clear indication of provider name and type; 1 – all other indications of ownership; zero – no indication of ownership); authorship (2 – clear indication of author name and credentials; 1 - all other indications of authorship; zero – no indication of authorship; source (2 – references related to scientific literature; 1 – all other sources; zero – no source indication); publication period (2 – clear indication of publication/updating date in all pages; 1 – all other indications of publication period; zero - no indication of publication period); interactivity (2 – clear invitation to post comments or ask questions via e-mail or link to web form; 1 – any other e-mail address listed on the website; zero – no interaction possibilities); web navigation (2 – information easily found on home page; 1 – information difficult to find via web links and search tools provided; zero – scattered information with no clear search mechanism); content balance (2 – balanced information; 1 – information biased towards proprietary products or services; zero – promotions limited to proprietary products or services). This is a 0-to-14 point score index. Websites scoring 5 or less, 6 to 10, or 11 to 14, were rated poor, average and excellent, respectively.

## RESULTS

**Figure 1:**
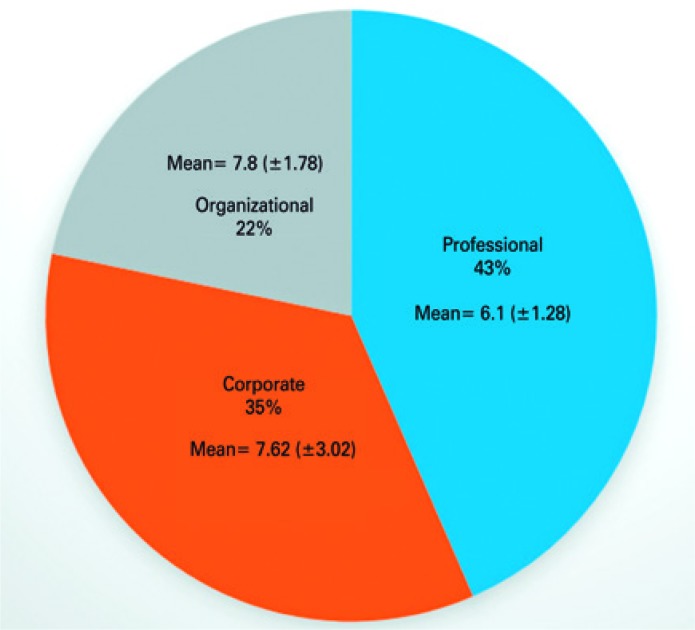
Average Sandvik Score according to website category

Twenty-three websites met the inclusion criteria, and were divided into three categories, as follows: professional (owned by universities, hospitals or clinics), organizational (owned by societies, foundations or scientific journals) and corporate (Figure 1).

**Figure 2:**
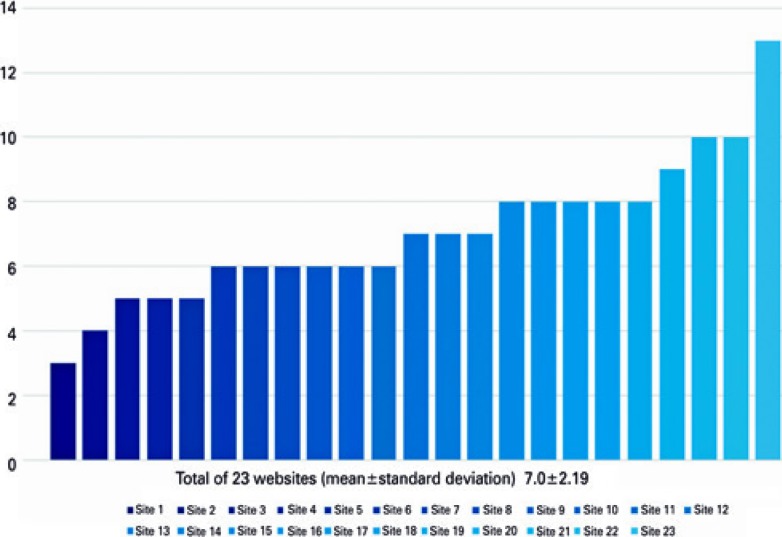
Final individual website scores (Sandvik Score)

Scores attributed to website quality (Sandvik Severity Index) ranged from 3 to 13 (Table 1), with a mean score of 7.0 (± 2.19) (Figure 2). A single website scored 13 and was rated excellent. Remaining websites were rated either average (17 web*s*ites; scores ranging from 6 to 10) or poor (5 websites; scores ranging from 3 to 5).

**Table 1:** Age-related macular degeneration assessment of sites

Sites	Sandvik Severity Index
http://www.minhavida.com.br/saude/temas/dmri	3
http://www.dayhorc.com.br/pt/saibamaissobre/degeneracao-macular/	4
http://www.cemahospital.com.br/degeneracao-macular-relacionada-a-idade-dmri/	5
http://www.iorj.med.br/degeneracao-macular-dmri/	5
https://pharma.bayer.com.br/pt/areas-terapeuticas/saude-de-a-a-z/degeneracao-macular/	5
http://vejaparasempre.com.br/sem-categoria/dmri/	6
http://www.cbo.net.br/novo/publico-geral/dmri.php	6
http://www.lotteneyes.com.br/patologias-degeneracao-macular-relacionada-a-idade/	6
http://www.ipvisao.com.br/site/especialidades-degeneracao_macular	6
http://www.botelho.med.br/especialidades/doencas-da-retina/degeneracao-macular-relacionada-a-idade	6
http://retinacuritiba.com.br/degeneracao-macular-tem-cura/	6
http://ceoportoalegre.com.br/2010/07/degeneracao-macular-relacionada-a-idade-dmri/	7
https://www.abcdasaude.com.br/oftalmologia/degeneracao-macular-relacionada-a-idade	7
https://www.criasaude.com.br/N13136/doencas/degeneracao-macular-relacionada-a-idade.html	7
http://www.scielo.br/scielo.php?script=sci_arttext&pid=S0034-72802010000600010	8
http://www.msdmanuals.com/pt/profissional/dist%C3%BArbios-oftalmol%C3%B3gicos/doen%C3%A7as-da-retina/degenera%C3%A7%C3%A3o-macular-relacionada-%C3%A0-idade-dmri	8
http://www.bausch.com.br/sua-saude/distubios-e-doencas/degeneracao-macular-relacionada-a-idade-dmri/	8
http://ioa.com.br/retina/degeneracao-macular-relacionada-idade/	8
http://www.saudebemestar.pt/pt/clinica/oftalmologia/degeneracao-macular/	8
http://retinabrasil.org.br/site/doencas/degeneracao-macular-relacionada-a-idade/	9
https://pt.wikipedia.org/wiki/Degenera%C3%A7%C3%A3o_macular_relacionada_%C3%A0_idade	10
https://drauziovarella.com.br/envelhecimento/degeneracao-da-macula-relacionada-a-idade-dmrj/	10
https://portal.novartis.com.br/o-que-e-dmri	13
Total of 23 websites (mean±standard deviation)	7,0±2,19

**Figure 3:**
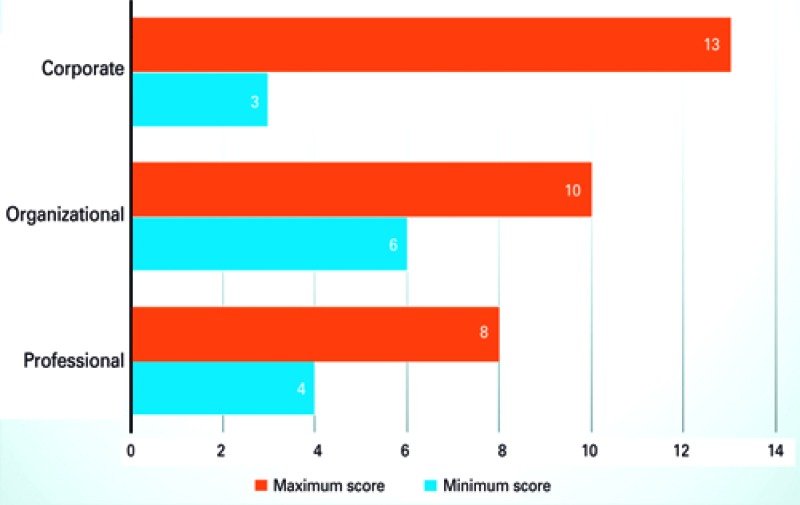
Sandvik Score variation per website category

Professional, organizational and corporate website scores ranged from 4 to 8 (mean score, 6.1±1.28), 6 to 10 (mean score, 7.80±1.78) and 3 to 13 (mean score, 7.62±3.02), respectively (Figure 3).

All websites in this sample scored full marks in ownership. References were listed in 30.43% of websites. Professional websites failed to indicate data sources and period of publication.

Seventeen websites scored full marks in web navigation and scored 15 in content balance. Website interactivity was poor overall (82.60% of websites achieving lowest scores in this item).

## DISCUSSION

Age-related macular degeneration is the leading cause of blindness in older adults living in industrialized countries, and its prevalence increases with age.^(^ ^1^ ^)^ The number of AMD cases is expected to rise in Brazil as life expectancy increases, in a clear reflection of social structural changes within the country.^(^ ^8^ ^)^

This was a pioneer study investigating the quality of online information about AMD available in Portuguese. Content was particularly emphasized to gauge website utility for the patient population in general, regardless of schooling level.

Internet access and related public health implications have been attracting increasing interest in Brazil. Del Giglio et al., investigated the quality of online information on three major diseases with high prevalence in the country (*diabetes mellitus*, hypertension and acute myocardial infarction). Different methods were used in that study; still, information available in Portuguese was often thought to be inappropriate and insufficient.^(^ ^14^ ^)^ In a study investigating the role of social media on dengue surveillance in Rio de Janeiro,^(^ ^15^ ^)^ Antunes et al., concluded this may be a major epidemiological tool in epidemic scenarios and a valuable strategy for epidemiological surveillance authorities.^(^ ^15^ ^)^

Websites investigated in this study were rated average according to the Sandvik Severity Index. This score was developed to measure urinary incontinence in women and evaluate related online information, and has been applied to different clinical conditions ever since.^(^ ^13^ ^)^ In a survey of 75 websites, Sandvik observed that most contained incomplete, albeit correct information on the disease.^(^ ^13^ ^)^ Gunasekera et al., investigated the quality of the most popular glaucoma-specific complementary and alternative medicine websites, and reported variable quality (9.4±2.6; Sandvik Score).^(^ ^16^ ^)^

Along with quality, Agi et al., also investigated readability and adequacy of general web-based information about glaucoma available in Portuguese. In that study, website quality was rated average and adequacy and readability were thought to be low.^(^ ^17^ ^)^

Other qualitative studies also investigated readability of internet-sourced material, which reflects the ability to understand and assimilate written information. Readability assessment is based on the Flesch-Kincaid Readability Test, designed to estimate the time (years of study) required to develop reading comprehension skills.^(^ ^18^ ^)^ Readability assessment confirmed a bias towards content aimed at a limited portion of the society, namely that with average to high sociocultural levels. Hence, a large proportion of people whose schooling levels are low would not be able to properly understand information disseminated on the websites included in that sample.^(^ ^18^ ^)^ Readability assessment also throws light on difficulties associated with analysis of information, as this tool was developed with the English language in mind and may therefore have limited applicability when translated into Portuguese. Also, educational systems differ between English-speaking countries and Brazil as regards the acquisition of reading comprehension skills. Therefore, readability was not included in this study.

The selection process in this study revealed a larger proportion of professional websites, most of which were owned by private clinical practices, with no attention given to data sources or publication dates. Two professional websites were rated poor. No organizational websites managed by societies/foundations/journals was rated excellent. In corporate websites, published information served advertising purposes and content was often neglected. Corporate website quality varied widely.

Sponsored websites tended to appear at the top of the match list, introducing a bias among web users. Also, most websites in this study were not academic and made excessive use of medical jargon (*e.g*., “neovascularization” and “drusen”). Another negative aspect was that the *Sociedade Brasileira de Oftalmologia* website did not come up often in web searches, even though it is a professional association.

The central idea behind this study was to investigate what actually happens when web search engines are used to obtain information about AMD. Should this sample have been limited to organizational websites, quality of information might have been better.

Another limiting factor was the highly dynamic nature of the internet, where information is constantly recycled. Web-based information and even selected websites themselves likely have changed since the beginning of this study.

The internet is an alternative source of information on health and diseases for those with limited access to health care.^(^ ^19^ ^)^ Age-related macular degeneration information available in Portuguese is apparently not aimed at the lay public, given the excessive use of technical jargon. Website content adjustment to a less technical level may enhance understanding of published data. Age-related macular degeneration information must therefore be reassessed for the sake of alignment with sociocultural standards of the Brazilian population in general.

Tools aimed to encourage user-author interaction (*e.g*., e-mail or chat) may also contribute to improve website quality.

Systematic, qualitative assessment of health-related written materials is vital to improve the current scenario. More strict control of general health information disseminated over the internet is a good alternative to improve website quality. Public policies aimed to improve education in Brazil and awareness raising campaigns promoted by professional associations, such as *Conselho Brasileiro de Oftalmologia* and *Sociedade Brasileira de Retina e Vítreo*, are thought to constitute major social change strategies.

## CONCLUSION

General information about age-related macular degeneration available on the internet was thought to be of average quality. These pieces of information are often disseminated by corporate websites owned by medical groups, with emphasis on medical product/treatment sales rather than content quality. The manner in which age-related macular degeneration data are presented on websites owned by professional associations should be revisited to enhance understanding by the general public. Insufficient quality control of internet-sourced health and disease information was highlighted, and the need for systematic reviews of online materials and introduction of more effective control mechanisms emphasized.
